# PSMB8 as a Core Target Mediating the Anti-Hepatocellular Carci-Noma Activity of Lingonberry (*Vaccinium vitis-idaea* L.) Extract in HepG2 Cells

**DOI:** 10.3390/cimb48030323

**Published:** 2026-03-18

**Authors:** Liangyu Zhu, Zhi Zhang, Yandong Zhang, Dianwen Wei, Zhenyu Wang, Liping Zhou

**Affiliations:** 1 Department of Natural Plant Functional Products, Heilongjiang Academy of Sciences Institute of Natural Resources and Ecology, Harbin 150040, China; liangyuzhu2025@163.com (L.Z.);; 2 Department of Forestry, Northeast Forestry University, Harbin 150040, China; 3 Department of Life Sciences and Food Engineering, Hebei University of Engineering, Handan 056038, China; 4 Department of Food Science and Engineering, Harbin Institute of Technology, Harbin 150090, China

**Keywords:** lingonberry extract, natural product, hepatocellular carcinoma (HCC), HepG_2_, PSMB8, apoptosis, xenograft model

## Abstract

Hepatocellular carcinoma (HCC) is a highly malignant tumour with a poor prognosis and few effective treatment options. Development of resistance to conventional therapies and occurrence of severe side effects highlight the urgent need for novel, low-toxicity interventions. Natural products are promising candidates for HCC drug development thanks to their multi-target activity and favourable safety profiles. Previous studies reported that Lingonberry extract, a bioactive natural product, inhibits proliferation of HepG_2_ cells. However, the key molecular targets and underlying anticancer mechanisms remain unclear. In this study, we analysed gene chip data from Lingonberry extract-treated HepG_2_ tumour-bearing mice using bioinformatics tools, employing a cross-species, multi-level screening strategy to identify PSMB8 as the core regulatory gene. In vitro functional validations (Western blotting, RT-PCR, CCK-8 assay, colony formation assay, flow cytometry and TUNEL staining) confirmed these findings. Downregulating PSMB8 was found to effectively induce late apoptosis in HepG_2_ cells, and Lingonberry extract was found to significantly reduce PSMB8 protein expression. This study identifies PSMB8 as a key mediator of the anticancer effect of Lingonberry extract in HepG_2_ cells. It provides a reliable methodological reference for screening anticancer targets of natural products and supports further exploration of Lingonberry extract as a potential adjuvant/lead compound for HCC.

## 1. Introduction

HCC is the most common type of primary liver cancer. It has become one of the most challenging malignancies worldwide [1,2]. Its incidence continues to rise, and the prognosis remains poor. Most patients are diagnosed at an advanced stage. They miss the opportunity for curative treatments such as surgery or local ablation.

The current standard of care for HCC involves multikinase and immune checkpoint inhibitors. These agents are typically given as part of combination regimens. These regimens regulate key oncogenic signalling pathways, including the Wnt/β-catenin, NF-κB, and PI3K/Akt/mTOR pathways [3,4]. Multikinase inhibitors primarily target downstream signals of receptor tyrosine kinases (e.g., VEGFR and EGFR), thereby inhibiting tumour angiogenesis and cell proliferation. Immune checkpoint inhibitors relieve T cell suppression to restore the body’s anti-tumour immune response [5,6]. Combination regimens of immunotherapy and targeted therapy have significantly improved patient prognosis. For instance, the combination of bevacizumab and atezolizumab extends overall survival in patients with advanced HCC. Regimens such as sorafenib combined with immunotherapy and lenvatinib plus PD-1 inhibitors have also demonstrated distinct clinical efficacy [7,8]. Despite these advances, however, HCC research still faces numerous challenges. Multikinase inhibitors are prone to primary or acquired resistance, which is often driven by mechanisms such as the reactivation of the NF-κB pathway [9]. Immune checkpoint inhibitors have limited objective response rates and are associated with immune-related toxicities [4]. Furthermore, the optimal treatment sequence and precise combination strategies for targeted therapies and immunotherapies remain to be elucidated. Additionally, access to standard care is limited for patients in developing countries. These unmet practical needs have led to the exploration of low-toxic, multi-target natural products. These natural products are potential adjuvant or lead agents for HCC intervention.

Plant-derived natural active components, including polyphenols, flavonoids, anthocyanins and phenolic acids, are a research focus for HCC intervention [10]. These components exhibit pharmacological properties such as multi-target synergistic regulation, low toxicity, and compatibility with other therapeutic modalities [11]. Numerous studies have confirmed that these natural active components exert anti-HCC effects. They act by regulating pathways associated with cell apoptosis, proliferation and metastasis, including the MAPK, PI3K/Akt/mTOR, and NF-κB pathways. Malvidin-3-galactoside, the primary anthocyanin found in blueberries, acts against HepG_2_ cells and tumour metastasis. This has been shown across in vitro and in vivo models. It inhibits cell proliferation, induces apoptosis and suppresses metastatic potential in these models. This activity is linked to PTEN/AKT pathway activation and the downregulation of MMP-2/9 expression [12,13]. Extracts from cranberries and blueberries, which are rich in diverse natural active components, significantly alleviate CCl_4_-induced liver fibrosis and injury. They do this by inhibiting inflammatory factors (IL-6, TNF-α) and reducing oxidative stress (MDA), thus providing evidence for their hepatoprotective potential in clinical applications [14]. Curcumin and resveratrol co-loaded nanoparticles exhibit significantly superior anti-HCC effects compared to the components individually, as they synergistically regulate apoptotic signalling pathways [15]. This indicates that multi-component natural active ingredients can avoid single-target resistance through complementary effects. They are therefore better suited to the long-term requirements of HCC intervention and offering new strategies to overcome therapeutic resistance in current anti-HCC regimens [16]. Moreover, plant active components can synergize with traditional targeted agents. For example, ellagic acid enhances the efficacy of sorafenib in killing HCC cells by blocking the MAPK/Akt/mTOR pathway [17]. This finding provides experimental evidence to support the optimisation of plant active components in combination with other agents for HCC suppression.

Lingonberry, a representative species of berries in the Vaccinium genus, is rich in polyphenolic components such as anthocyanins, flavonoids, and phenolic acids. The chemical stability and bioavailability of its extract have been verified [18,19,20,21], supporting its potential application as a complementary intervention agent. In terms of anti-cancer activity, Lingonberry extract exhibits broad-spectrum inhibitory effects. It can significantly inhibit the proliferation of colorectal (HT-29), malignant melanoma (IGR39), and hepatocellular carcinoma (HepG_2_) cells [22,23]. Notably, Lingonberry extract has a clear hepatoprotective effect. In models of non-alcoholic fatty liver disease, it alleviates liver injury by reducing hepatic lipid accumulation, inhibiting oxidative stress, and suppressing NF-κB-mediated inflammatory responses [24]. It can also target the Notch1 signalling pathway to regulate hepatic lipid metabolism [25]. This property addresses the key issue of hepatotoxicity associated with therapeutic agents, providing direct experimental evidence for the safety of lingonberry extract as a potential adjuvant intervention against HCC.

Notably, PSMB8, a subunit of the immunoproteasome, has emerged as a key target associated with HCC progression. PSMB8 is a critical member of the PSM family and is abnormally overexpressed in HCC tissues. It is closely linked to tumour malignancy and poor patient prognosis. PSMB8 drives HCC progression by promoting cell proliferation, migration, and epithelial–mesenchymal transition (EMT). Its oncogenic effects are mediated by the upstream transcriptional regulation of ZNF655. They are also closely associated with the activation of the PI3K/AKT oncogenic pathway [26]. Furthermore, dysregulated PSMB8 expression contributes to genomic instability and the maintenance of cancer stem cell properties in HCC [27]. Collectively, these findings underscore the potential of PSMB8 as a promising therapeutic target for HCC. However, the regulatory effects of natural plant bioactive components on PSMB8 in HCC cells remain unclear. The underlying molecular mechanisms also require further investigation.

In our previously published work, we confirmed that Lingonberry extract can inhibit the proliferation of HepG_2_ hepatoma cells in vitro [23]. Building on this observation, we further verified the anti-tumour effect of the extract in HepG_2_ tumour-bearing mouse models. However, the key molecular targets that mediate the anti-tumour activity of Lingonberry extract in HepG_2_ cells remain unclear. The potential regulatory relationship between Lingonberry extract and PSMB8 in this cell line has also not been elucidated. To identify novel targets of Lingonberry extract in HCC intervention and clarify its correlation with PSMB8, we performed bioinformatics analysis. This analysis was on gene expression data from HepG_2_ tumour-bearing mice treated with Lingonberry extract. Further in vitro functional validation was then conducted in HepG_2_ cells. PSMB8 was selected as a candidate core regulatory gene through bioinformatics screening. Subsequent in vitro experiments confirmed that PSMB8 mediates the anti-HepG_2_ activity of Lingonberry extract. This study provides a novel perspective on the anti-tumour mechanism of Lingonberry extract in HepG_2_ cells. It also offers theoretical support and experimental evidence for the development of Lingonberry extract as a potential adjuvant intervention or natural product-derived lead compound for HCC.

## 2. Materials and Methods

### 2.1. Materials and Reagents

Lingonberry fruits were purchased from Tahe County, Daxing’anling, China, and stored at −20 °C as fresh frozen samples until use. The extraction, purification, and component characterization of Lingonberry extract were performed exactly as described in our previously published study [23]. Briefly, the extract powder was obtained via ethanol extraction, macroporous resin (YWD07) and polyamide resin purification, and freeze-drying. HPLC-based component characterization, key component quantification and batch-to-batch functional consistency verification by CCK-8 assay are detailed in Supplementary Material S1.

Prior to the experiment, the lyophilized extract was dissolved in normal saline to the desired concentrations and filtered through a 0.22 μm membrane for sterilisation.

The HepG_2_ cell line used in the animal experiment and in vitro validation was a commercially available product purchased from Shanghai Jinyuan Biotechnology Co., Ltd. Shanghai, China (official website: http://www.ssrcc.com.cn/ accessed on 1 June 2025). Cell lines were confirmed to be mycoplasma-free and authenticated via short tandem repeat (STR) genotyping within the past three years, with identity verified against STR profiles in comparable databases (Zhejiang Ruyao Biotechnology Co., Ltd., Taizhou, China).

High-glucose Dulbecco’s Modified Eagle Medium (DMEM) was purchased from Gibco (Thermo Fisher Scientific, Waltham, MA, USA). Foetal bovine serum (FBS) was obtained from Invitrogen (Thermo Fisher Scientific, Waltham, MA, USA). Penicillin-streptomycin solution and phosphate-buffered saline (PBS) were sourced from Sigma-Aldrich (St. Louis, MO, USA) and Gibco (Waltham, MA, USA), respectively. 0.25% Trypsin was acquired from HyClone (Cytiva, Logan, UT, USA). 6-well, 24-well, and 96-well tissue culture plates were purchased from Corning (Corning, NY, USA). Cell Counting Kit-8 (CCK-8) was obtained from Dojindo Laboratories (Kumamoto, Japan). Annexin V-FITC/Propidium Iodide (PI) apoptosis assay kits were supplied by Beyotime Biotechnology (Shanghai, China).

### 2.2. Cell Culture

HepG_2_ cells were cultured in DMEM supplemented with 10% FBS at 37 °C in a humidified atmosphere containing 5% CO_2_.

### 2.3. Animal Experiments

The animal experiment was conducted as part of the first author’s doctoral dissertation (2022, DOI: 10.27009/d.cnki.gdblu.2022.001858. It can be retrieved by searching this DOI on the CNKI website (https://www.cnki.net/ accessed on 1 June 2025). Detailed data from the animal experiment are included in the data set. The tumor tissues from this experiment were used for transcriptome sequencing in the present study. All procedures were performed in accordance with the National Institutes of Health Guide for the Care and Use of Laboratory Animals and approved by the Institutional Animal Care and Use Committee (IACUC) of Harbin Institute of Technology (Approval No. IACUC-2019017).

Forty male BALB/c nude mice (4-week-old, 18–20 g) were purchased from Beijing Weitalihua Experimental Animal Technology Co., Ltd (Beijing, China). and housed under specific pathogen-free (SPF) conditions (22 ± 1 °C, 50% humidity, 12 h light/dark cycle) with free access to sterile food and water. Six of the 40 mice were designated as the normal control group without any cell inoculation (the normal control group was also gavaged with normal saline, consistent with the model group’s intervention route). HepG_2_ cells (5 × 10^5^ cells in 100 μL PBS) were subcutaneously injected into the right flank of the remaining 34 mice to establish tumour models. Ten days after inoculation, mice with successful tumour formation (tumour volume ≥ 50 mm^3^) were randomly divided into 5 groups (*n* = 6/group): model group (gavaged with normal saline), low/medium/high-dose Lingonberry extract groups (50/100/200 mg/kg, daily gavage), and positive control group (cyclophosphamide 30 mg/kg, alternate-day gavage).

After 28 consecutive days of intervention, all mice were euthanized by cervical dislocation to minimise suffering (no animals reached humane endpoints such as tumour volume >2000 mm^3^ or >20% weight loss during the experiment). Tumours were excised on ice, snap-frozen in liquid nitrogen, and tissues from the high-dose group (with the most significant anti-tumour effect confirmed in the doctoral dissertation) and model group were selected for transcriptome sequencing.

### 2.4. Gene Chip Detection Methodology

Tumour tissues from the tumour model group and Lingonberry group were dissected. Total RNA was isolated and submitted for detection using the DNBSEQ platform. BGI (Beijing Genomics Institute, Beijing, China) performed all sequencing-related procedures according to their standard operating protocols (SOPs). These included RNA quality control, library construction, and sequencing on the DNBSEQ platform.

The samples consisted mainly of human hepatocellular carcinoma cells, with a small proportion of mouse stromal cells. To avoid losing key regulatory signals from host contamination during alignment, initial differential gene analysis was performed. The reference genome used was *Mus musculus* (GCF_000001635.26_GRCm38.p6), which was obtained from the NCBI database.

Raw sequencing data were first subjected to quality control using BGI’s internal filtering pipeline. Clean reads were then aligned to the mouse reference genome, and a second round of quality control was conducted. Following quality validation, the data were used for downstream bioinformatic analysis. Gene expression counts and related information are provided in Supplementary Materials S2 and S3.

### 2.5. Bioinformatics Analysis and Screening Strategy

**Figure 1:**
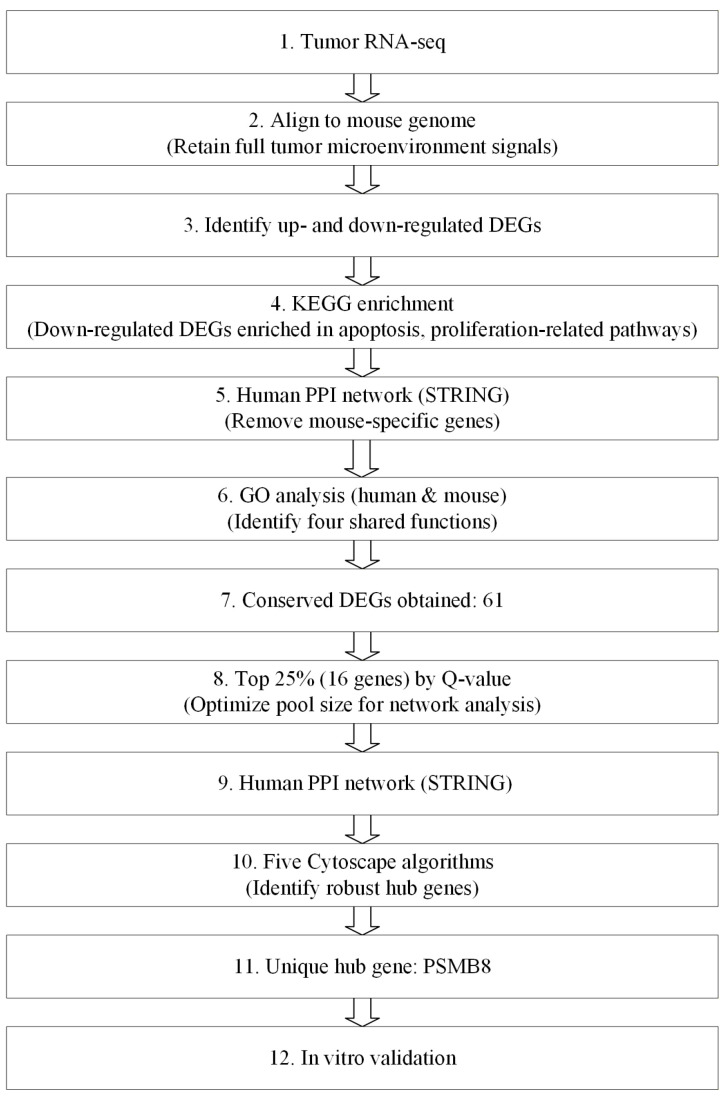
Schematic overview of the bioinformatics pipeline.

The overall bioinformatics analysis workflow is illustrated in Figure 1.

To address the research aim of this study, we established a dedicated bioinformatics analysis and screening strategy. This strategy prioritised evolutionarily conserved genes shared between human and mouse, rather than species-specific genetic elements.

In conventional species-disambiguation pipelines, some functionally important conserved genes are frequently lost. This is mainly caused by the mixed cell population in tumour tissues, which contains both host (mouse) and human cells. During analysis, such heterogeneity can lead to overly strict filtering or artefacts from cross-species contamination. These factors may cause key conserved genes to be incorrectly excluded.

Our strategy was designed to avoid this issue and retain a relatively more complete set of evolutionarily conserved genes. Briefly, raw reads were aligned to the mouse reference genome (GCF_000001635.26 GRCh38.p6). This step helped remove human-specific genes that cannot be mapped. Subsequent filtering using the human PPI network was applied to exclude mouse-specific genes. This process yielded a set of conserved genes shared between the two species. This set was further refined by GO biological process (BP) enrichment analysis and topological analysis in the PPI network. This step allowed us to identify core target genes. These genes were then validated by in vitro experiments. Although this pipeline carries a theoretical risk of cross-species read misassignment, it does not affect our core objective. Mapping reliability from sequence alignment alone is also limited. However, these factors do not influence the identification of functionally conserved genes shared by human and mouse. Moreover, potential uncertainty from sequence mapping was minimised by topological filtering. It was further excluded by in vitro validation.

KEGG pathway enrichment analysis was performed on differentially expressed genes (DEGs). DEGs associated with apoptosis and tumour-suppressive pathways were extracted and submitted to the STRING human PPI database (v10.5). The interaction confidence threshold was dynamically set to 0.7 for gene sets of 20 or fewer genes to ensure high-confidence interactions during hub identification. For gene sets containing more than 20 genes, the threshold was adjusted to 0.4 to retain enough candidates for cross-species comparison.

GO-BP analysis was conducted using both human and mouse databases. The top 5 significant terms from each species were compared, and 4 shared functional terms were identified. Genes corresponding to these common terms were intersected, yielding 61 conserved DEGs.

Genes were ranked by adjusted Q-value in descending order of significance. The top 25% (16 genes) were selected as the core gene pool for hub screening. This threshold was empirically optimized to balance statistical significance and network connectivity. A more stringent cutoff would reduce the gene number excessively, preventing reliable topological analysis. A more relaxed cutoff would introduce non-essential genes and dilute the identification of true functional hubs.

Core target identification was performed using 5 independent analytical methods in Cytoscape (v3.9.0). Molecular Complex Detection (MCODE) was used to identify densely connected network modules. The highest-scoring module, containing 10 genes including PSMB8, was selected. Four topological algorithms, including Maximal Neighborhood Component (MNC), Betweenness, Bottleneck, and Clustering Coefficient, were used to evaluate gene centrality. For each algorithm, the eight most influential nodes were selected from the core pool of 16 genes. PSMB8 was identified as the final core target. It was the only gene included in all five independent screening lists, confirming its stable and non-random centrality within the network.

Venn diagrams were generated using the OmicShare platform (https://www.omicshare.com/tools accessed on 1 June 2025). Enrichment analysis was performed using the hypergeometric test in R, with a significance threshold of Q-value ≤ 0.05.

Reference genomes: *Homo sapiens:* GCF_000001405.39_GRCh38.p13; *Mus musculus:* GCF_000001635.26_GRCm38.p6.

### 2.6. Lentiviral Packaging Transfection of HepG_2_ Cells and Grouping

The cDNA sequence encoding PSMB8 was digested with BamH I and Apa I, then cloned into the pLVX-CMV-MCS-Puro lentiviral vector using T4 DNA ligase. For PSMB8 knockdown, siRNA-targeting oligos were designed, annealed to form double-stranded DNA, and ligated into the EcoRI/AgeI-digested pLKO.1 vector to generate pLKO.1-shRNA recombinant lentivirus. The constructed lentiviral vectors (or empty vectors) were co-transfected with psPAX2 and pMD2. G packaging plasmids into 293T cells. The viral supernatant was collected 72 h post-transfection, filtered, and added to HepG_2_ cells. Infections were allowed to proceed for 24 h.

PSMB8 experiment grouping: Empty PSMB8 group, PSMB8 Vector group, Control ShRNA group, PSMB8 shRNA group.

PSMB8 regulation by Lingonberry extract: Control group, Extract group (The optimal effective concentration of Lingonberry extract, verified by the CCK-8 assay, was determined to be 70 μg/mL. See Supplementary Material S1 for details), OE-PSMB8 group (PSMB8 overexpression), Extract+OE-PSMB8 (PSMB8 overexpression + 70 μg/mL Lingonberry extract treatment).

### 2.7. PSMB8 Expression Analysis in Cancer Cells by Western Blotting

Protein extraction from HepG_2_ cells in each group was performed using RIPA lysis buffer. Protein concentration was quantified with a BCA kit (Thermo Fisher Scientific, Waltham, MA, USA). Protein samples were separated by SDS-PAGE and transferred to PVDF membranes, which were blocked with 5% non-fat milk at room temperature for 1 h. Membranes were incubated with primary antibodies at 4 °C overnight, washed with TBST, and then probed with secondary antibodies for 2 h at room temperature. After three TBST washes, chemiluminescent detection was performed in a darkroom, followed by film development and fixation. Film images were analysed using Quantity One gel analysis software (v4.6.2). Densitometric quantification of the Western blotting bands was performed using ImageJ software (v1.53). Band intensities were normalized to the internal reference protein GAPDH.

### 2.8. PSMB8 Expression in Cancer Cells by RT-PCR

Total RNA was extracted and validated, followed by cDNA synthesis in 0.2 mL RNase-free EP tubes using the following reaction conditions: 15 min at 37 °C, 5 s at 85 °C, and 60 min at 4 °C. The synthesised cDNA was diluted with 180 μL of ddH_2_O for subsequent second-strand cDNA synthesis or PCR amplification and stored at −20 °C.

Primer sequences for quantitative PCR were designed as follows:

GAPDH-F: 5′-GTCTCCTCTGACTTCAACAGCG-3′

GAPDH-R: 5′-ACCACCCTGTTGCTGTAGCCAA-3′

PSMB8-F: 5′-GCTGCCTTCAACATAACATCA-3′

PSMB8-R: 5′-CTGCCACCACCACCATTA-3′

The PCR program was set up for amplification and relative expression levels were calculated using 2^−ΔΔCt^.

### 2.9. Cell Viability Assay via CCK-8 Method

HepG_2_ cells from the control and transfected groups were seeded into 96-well plates at 1 × 10^4^ cells/well and incubated for 4 h at 37 °C in a humidified 5% CO_2_ atmosphere. After cell attachment, the medium was discarded, and each well was supplemented with 100 μL of fresh medium and 10 μL of CCK-8 reagent (from Dojindo Laboratories, Kumamoto, Japan). Following a 2 h incubation at 37 °C, absorbance values were measured at 490 nm using a microplate reader.

### 2.10. Colony Formation Assay

HepG_2_ cells from the control and transfected groups were seeded into 6-well plates at a density of 1 × 10^3^ cells/well with three technical replicates per group. Cells were cultured at 37 °C with medium changes every 3 days. Visible colonies were typically observed after ~2 weeks. Following incubation, the medium was discarded, and cells were washed thrice with PBS, fixed with 4% paraformaldehyde for 15 min, and stained with 0.1% crystal violet solution for 20 min. After washing with PBS and air-drying, colonies were counted under an inverted microscope and photographed.

### 2.11. Cell Apoptosis Analysis by Flow Cytometry

HepG_2_ cells from each experimental group were seeded into 24-well plates at a density of 2 × 10^5^ cells/well and cultured for 24 h at 37 °C in a humidified 5% CO_2_ incubator. Following trypsinization with 0.25% trypsin, cells were washed twice with ice-cold PBS and resuspended in 1× Binding Buffer at a concentration of 1 × 10^6^ cells/mL. Single-stained compensation controls (Annexin V-FITC only and PI only) were prepared from untreated HepG_2_ cells to adjust for spectral overlap between fluorophores. For staining, 100 μL of cell suspension was transferred to a flow cytometry tube, followed by the addition of 5 μL Annexin V-FITC and 5 μL PI. The mixture was gently vortexed and incubated for 10–15 min at room temperature in the dark. Flow cytometry analysis was performed within one hour.

A standard gating strategy was applied to exclude debris and cell aggregates using forward scatter (FSC) vs. side scatter (SSC) plots, selecting a pure single-cell population for analysis. Quadrant interpretation was defined as follows: lower left (LL) quadrant, viable cells (Annexin V^−^/PI^−^); lower right (LR) quadrant, early apoptotic cells (Annexin V^+^/PI^−^); upper right (UR) quadrant, late apoptotic cells (Annexin V^+^/PI^+^); upper left (UL) quadrant, necrotic cells (Annexin V^−^/PI^+^). The total apoptotic rate was calculated as the sum of early (LR) and late (UR) apoptotic cells.

### 2.12. TUNEL Assay for Apoptosis Detection

Apoptosis was quantified using the One Step TUNEL Apoptosis Assay Kit (supplied by Beyotime Biotechnology, Shanghai, China) following the manufacturer’s instructions. Briefly, cells were fixed with 4% paraformaldehyde for 30–60 min at room temperature, washed twice with PBS, and permeabilized with 0.1% Triton X-100 in PBS on ice for 2 min. Following permeabilization, cells were incubated with 50 μL of TUNEL Reaction Mixture at 37 °C in the dark for 1 h. After three washes with PBS, nuclei were counterstained with DAPI for 5 min at room temperature in the dark. Samples were then washed three times with PBS and mounted using an anti-fade mounting medium. Fluorescence images were captured using a confocal microscope, and apoptotic cells were quantified by counting TUNEL-positive nuclei as a percentage of total DAPI-stained nuclei.

### 2.13. Statistical Analysis

All experiments were conducted using SPSS Statistics 17.0. One-way analysis of variance (ANOVA) followed by Fisher’s Least Significant Difference (LSD) post hoc test was used to evaluate significant differences between multiple groups. Statistical significance was defined as *p* ≤ 0.05 (*) and high significance as *p* ≤ 0.01 (**). All cellular data are presented as the mean ± standard deviation (SD) from at least three independent experiments.

## 3. Results

### 3.1. Differential Gene Expression Analysis Between Lingonberry Group and Tumour Model Group

**Figure 2:**
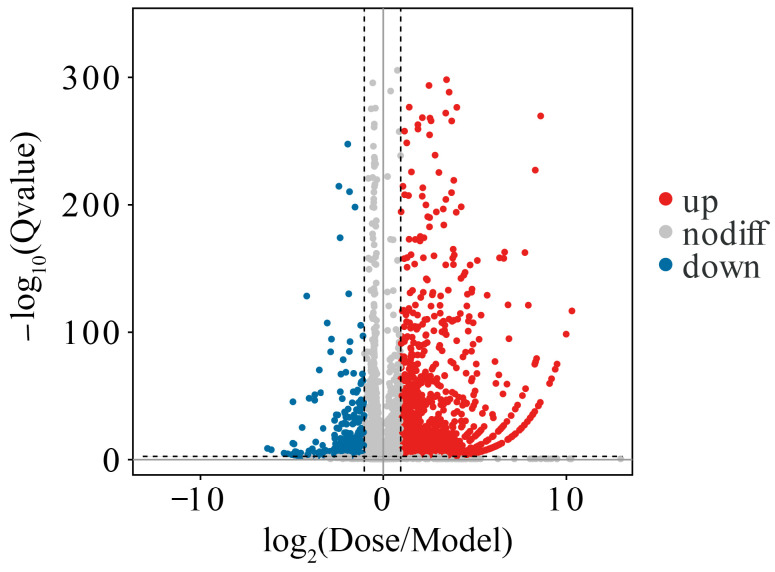
Differential gene expression analysis between Model Group and Dose Group. Volcano plot illustrating DEGs, with red dots representing upregulated genes and blue dots downregulated genes.

Using the *Mus musculus* reference genome, we analysed the gene expression profiles of the tumour model group (Model Group) and the Lingonberry group (Dose Group). A total of 1686 significantly DEGs were identified. Compared to the Model Group, the Dose Group had 1410 upregulated genes and 276 downregulated genes. The overall distribution characteristics of these DEGs are illustrated in a volcano plot (Figure 2).

### 3.2. KEGG Pathway Analysis of Differentially Expressed Genes

**Figure 3:**
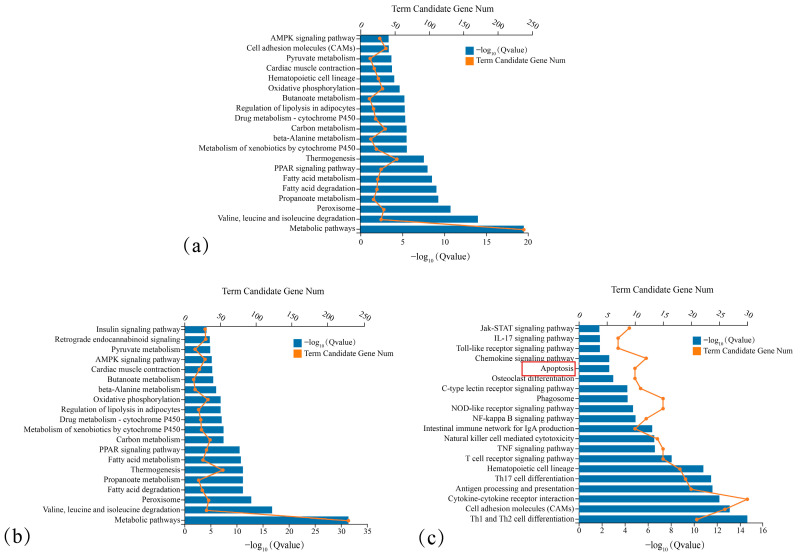
KEGG pathway annotation of DEGs between Model Group and Dose Group. (**a**) Top enriched pathways for total DEGs. (**b**) Top enriched pathways for upregulated DEGs. (**c**) Top enriched pathways for downregulated DEGs. The red box highlights the Apoptosis pathway, the key enriched term for downregulated DEGs.

KEGG pathway enrichment analysis was performed for all identified DEGs, as well as for upregulated and downregulated DEGs separately. As shown in Figure 3, downregulated DEGs were significantly enriched in apoptosis-related signalling pathways among the top 20 enriched terms. This enrichment was more prominent than that observed in all DEGs or upregulated DEGs. In addition, downregulated DEGs were significantly enriched in multiple cell proliferation-related pathways, including the Jak-STAT, NF-κB and TNF pathways. These results suggest a close association between downregulated DEGs, the inhibition of cancer cell proliferation, and the promotion of cancer cell apoptosis. Thus, downregulated DEGs were selected as the target gene set for subsequent analyses.

### 3.3. Core Gene Analysis of Downregulated Differentially Expressed Genes

**Figure 4:**
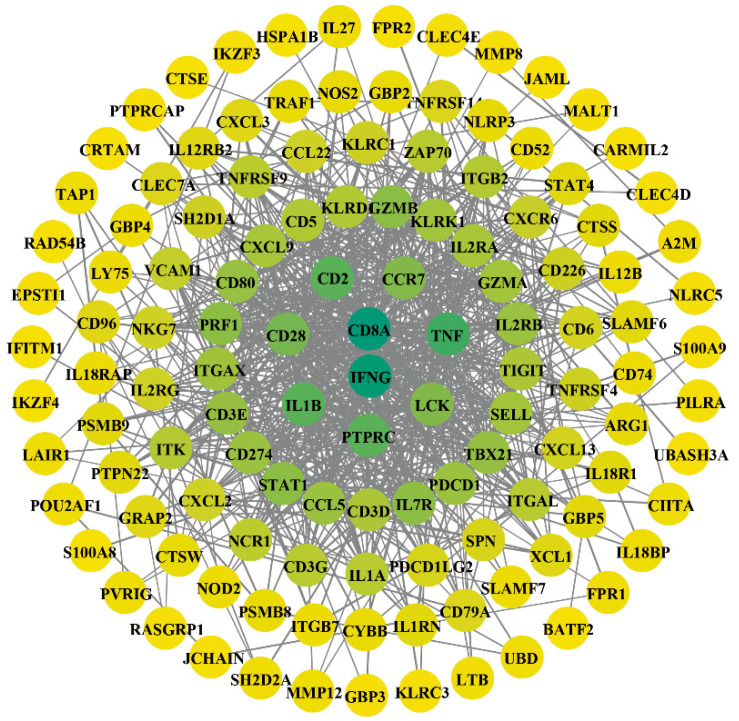
PPI network of downregulated DEGs. Nodes are colored on a gradient from yellow to green, reflecting an increase in protein-protein interaction (PPI) strength from low to high.

Downregulated DEGs linked to apoptosis and tumour-suppressive pathways (based on the mouse reference genome) were submitted to the human STRING PPI database. Mouse-specific genes were excluded, yielding DEGs that were downregulated in both humans and mice. A PPI network was then constructed (Figure 4). This network shows the interactions among the conserved genes. It provides a core gene set for subsequent functional enrichment and hub identification analyses.

**Figure 5:**
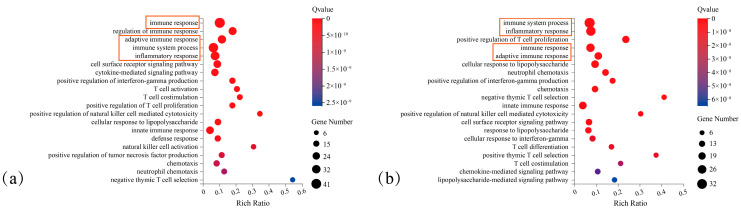
GO-BP functional annotation of downregulated DEGs. (**a**) Human (*Homo sapiens*) reference genome annotation. (**b**) Mouse (*Mus musculus*) reference genome annotation. The red boxes highlight the identical functional terms shared by murine and human sources.

Shared downregulated DEGs were subjected to GO-BP analysis separately in mice and humans. The results identified four common terms among the top five most significantly enriched functional terms of both species (Figure 5).

**Figure 6:**
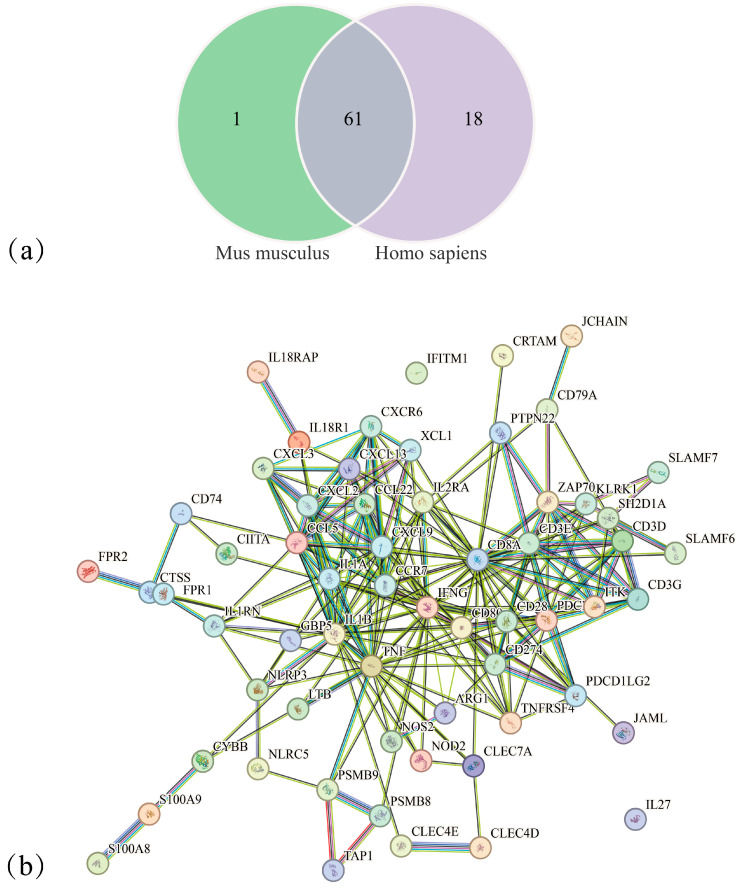
PPI network of mouse-human orthologous genes with identical nomenclature. (**a**) Venn diagram of shared orthologous genes between mouse and human. (**b**) PPI network of orthologous genes based on human proteome. Node colors in the PPI network indicate automatically generated functional clusters, where similar colors represent proteins belonging to the same functional module.

The four terms were immune response, adaptive immune response, immune system process, and inflammatory response. These terms corresponded to 62 DEGs in mice and 79 DEGs in humans. Sixty-one shared DEGs were obtained by intersecting the gene lists from both species. PPI analysis was then performed for these 61 DEGs using the human genome as the reference (Figure 6).

**Figure 7:**
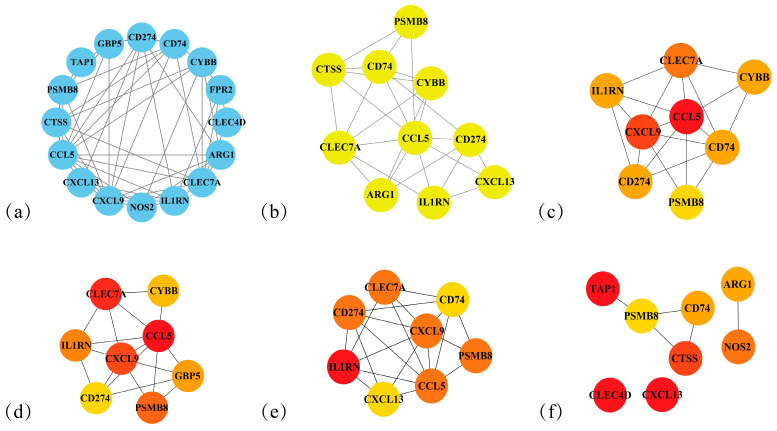
Key downregulated DEGs between Model Group and Dose Group. (**a**) Top 16 significantly differentially expressed genes. (**b**) MCODE analysis results. (**c**) MNC scores. (**d**) Betweenness centrality values. (**e**) Bottleneck centrality analysis. (**f**) Clustering coefficient distribution. (**c**–**f**) The color gradient from yellow to red indicates increasing scores (darker red represents higher scores).

To further refine candidate gene selection, the 61 DEGs were ranked in descending order of differential significance based on Q-value (Supplementary Material S3). The top 25% of genes were selected to form a core gene pool, which contained 16 DEGs in total. The human PPI network of this core pool is shown in Figure 7a.

**Figure 8:**
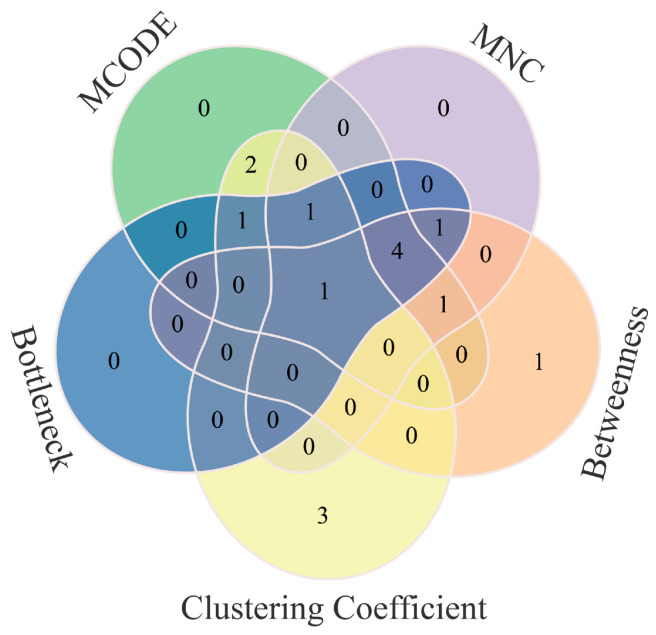
Core hub gene identification. Each color represents a distinct topological analysis method (MCODE, MNC, Bottleneck, Betweenness, Clustering Coefficient), with color shading variations indicating overlapping regions between methods and no additional biological significance.

Based on this network, five algorithms were applied to screen for core targets (Figure 7). These included MCODE, MNC, Betweenness, Bottleneck, and Clustering Coefficient. Cross-analysis of the outputs from all five algorithms identified PSMB8 as the key hub gene (Figure 8).

### 3.4. Effects of PSMB8 on Cancer Cell Proliferation and Apoptosis

#### 3.4.1. PSMB8 Transfection Efficiency Validation

**Figure 9:**
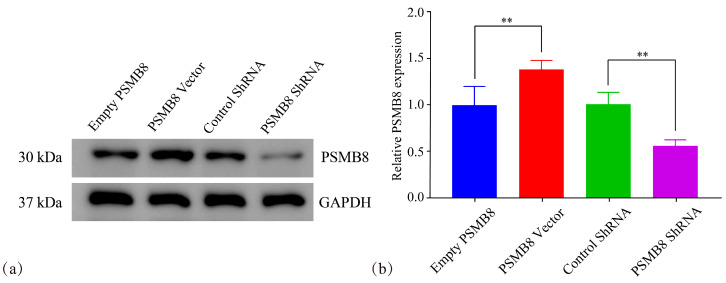
Western blotting analysis and densitometric quantification of PSMB8 protein expression in HepG_2_ cancer cells. (**a**) Representative Western blotting bands of PSMB8 and GAPDH. (**b**) Densitometric quantification of PSMB8 protein levels normalized to GAPDH. Data are presented as mean ± SD from three independent biological replicates (*n* = 3). Statistical analysis was performed using one-way ANOVA followed by LSD post-test. ** *p* ≤ 0.01.

Western blotting analysis showed that PSMB8 protein expression was significantly higher in the PSMB8 overexpression group (PSMB8 Vector) than in the empty vector control group (Empty PSMB8). In contrast, PSMB8 protein levels were markedly decreased in the PSMB8 shRNA interference group (PSMB8 shRNA) compared with the negative control shRNA group (Control shRNA). Densitometric quantification of the Western blotting bands further confirmed these differential expression patterns (Figure 9).

**Figure 10:**
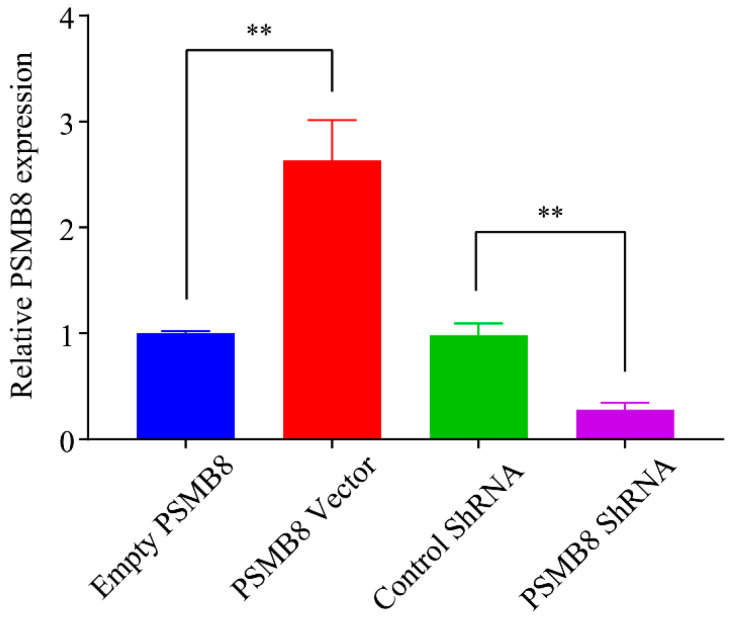
RT-PCR quantification of PSMB8 mRNA expression in HepG_2_ cancer cells. Data are presented as mean ± SD from three independent biological replicates (*n* = 3). Statistical analysis was performed using one-way ANOVA followed by LSD post-test. ** *p* ≤ 0.01.

RT-PCR analysis further confirmed increased PSMB8 mRNA levels in the overexpression group and efficient knockdown of PSMB8 mRNA in the interference group (Figure 10). These results indicate successful modulation of PSMB8 expression by lentiviral transfection.

#### 3.4.2. PSMB8 Modulates HepG_2_ Cell Proliferation

To investigate the regulatory role of PSMB8 in HepG_2_ cell proliferation, cell viability was measured at 24 and 48 h. The empty PSMB8 group was set as 100% cell viability. The PSMB8 Vector group significantly increased cell viability to 147.47% ± 4.84 at 24 h and 157.48% ± 9.86 at 48 h. These values were highly significantly higher than those of the Empty PSMB8 group (*p* ≤ 0.01).

The Control shRNA group was used as the 100% viability baseline. The PSMB8 shRNA group reduced cell viability to 63.93% ± 3.40 at 24 h and 31.8% ± 5.53 at 48 h. These values were highly significantly lower than those of the Control shRNA group (*p* ≤ 0.01).

**Figure 11:**
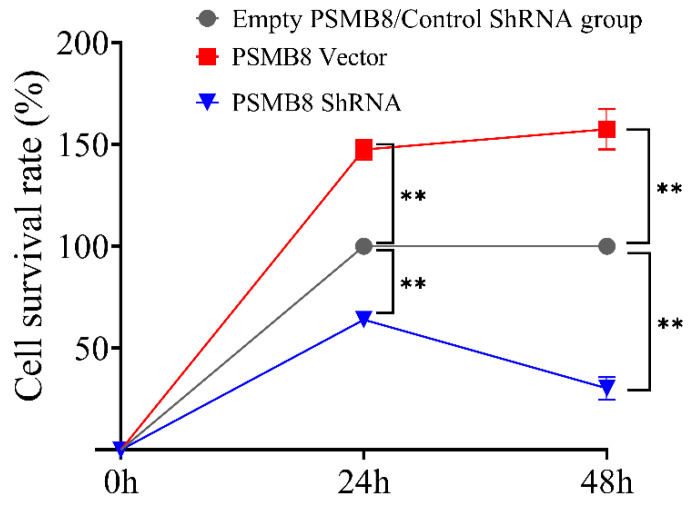
Growth curve of HepG_2_ cells in PSMB8 intervention groups. Data are presented as mean ± SD from three independent biological replicates (*n* = 3). Statistical analysis was performed using one-way ANOVA followed by LSD post-test. ** *p* ≤ 0.01.

The PSMB8 Vector group displayed highly significantly higher viability than the PSMB8 shRNA group at both time points. PSMB8 overexpression highly significantly promoted HepG_2_ cell proliferation. Inhibition of PSMB8 expression effectively suppressed cell proliferation. This regulatory effect was strengthened with prolonged culture time (Figure 11).

**Figure 12:**
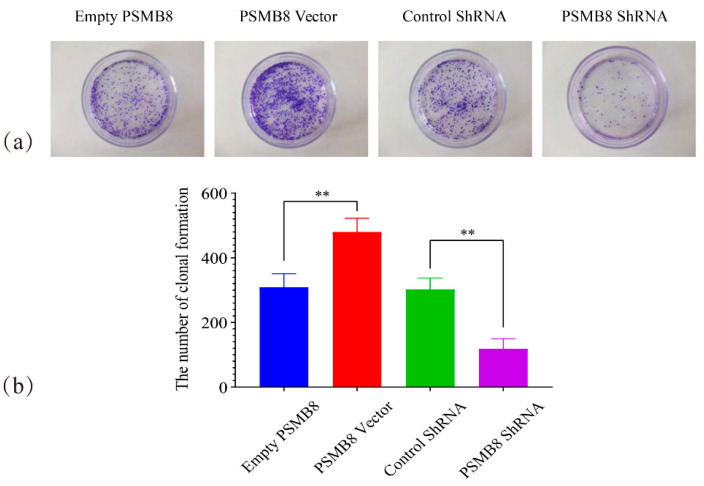
Effect of PSMB8 expression on HepG_2_ cell clonogenicity. (**a**) Representative images of cell clones in each PSMB8 experimental group. (**b**) Quantitative analysis of clonogenic capacity. Data are presented as mean ± SD from three independent biological replicates (*n* = 3). Statistical analysis was performed using one-way ANOVA followed by LSD post-test. ** *p* ≤ 0.01.

Further verification was performed using a colony formation assay (Figure 12). The PSMB8 Vector group formed 480 ± 43 colonies. This number was highly significantly higher than that in the Empty PSMB8 group (309 ± 42 colonies, *p* ≤ 0.01). The PSMB8 shRNA group formed 119 ± 31 colonies. This number was highly significantly lower than that in the Control ShRNA group (302 ± 35 colonies, *p* ≤ 0.01). Consistent results from both assays confirmed that PSMB8 overexpression significantly promoted HepG_2_ cell proliferation. Inhibition of PSMB8 expression effectively suppressed cell proliferation.

### 3.5. Effect of PSMB8 Expression on Apoptosis in HepG_2_ Cells

**Figure 13:**
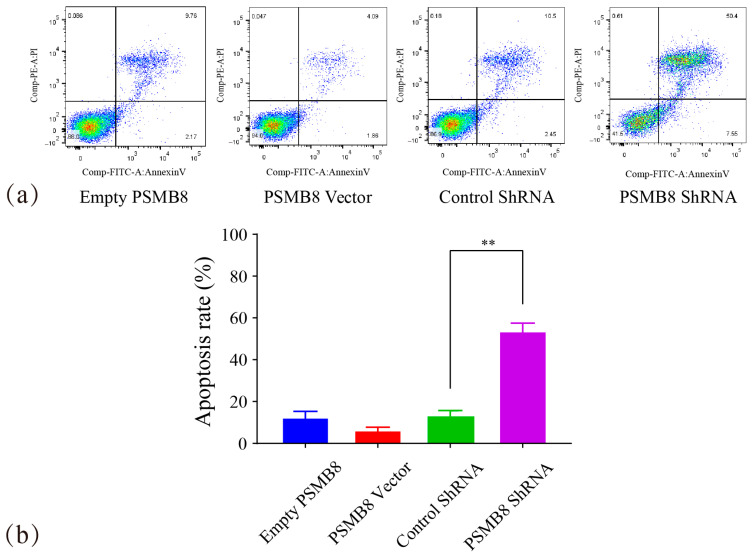
Effect of PSMB8 expression on HepG_2_ cell apoptosis. (**a**) Flow cytometry analysis of apoptosis in each PSMB8 experimental group. In the flow cytometry dot plots, green fluorescent signals represent Annexin V-FITC staining and blue signals denote PI staining; color clusters distinguish cells at different apoptotic stages. (**b**) Quantitative analysis of apoptotic cells. Data are presented as mean ± SD from three independent biological replicates (*n* = 3). Statistical analysis was performed using one-way ANOVA followed by LSD post-test. ** *p* ≤ 0.01.

To investigate the regulatory role of PSMB8 in HepG_2_ cell apoptosis, flow cytometry was used. Apoptotic rates and apoptotic stage distribution were analysed, and the results are shown in Figure 13.

The apoptotic rate of the PSMB8 Vector group was 5.96% ± 1.74. This value was lower than that of the Empty PSMB8 group (11.8% ± 3.45). The apoptotic rate of the PSMB8 shRNA group was 53% ± 4.47. This value was highly significantly higher than that of the Control shRNA group (12.9% ± 2.81, *p* ≤ 0.01).

Apoptotic cells were mainly distributed in the late apoptotic stage. These data indicated that inhibition of PSMB8 expression promoted HepG_2_ cell apoptosis. This effect was mediated by the induction of the late apoptotic pathway.

**Figure 14:**
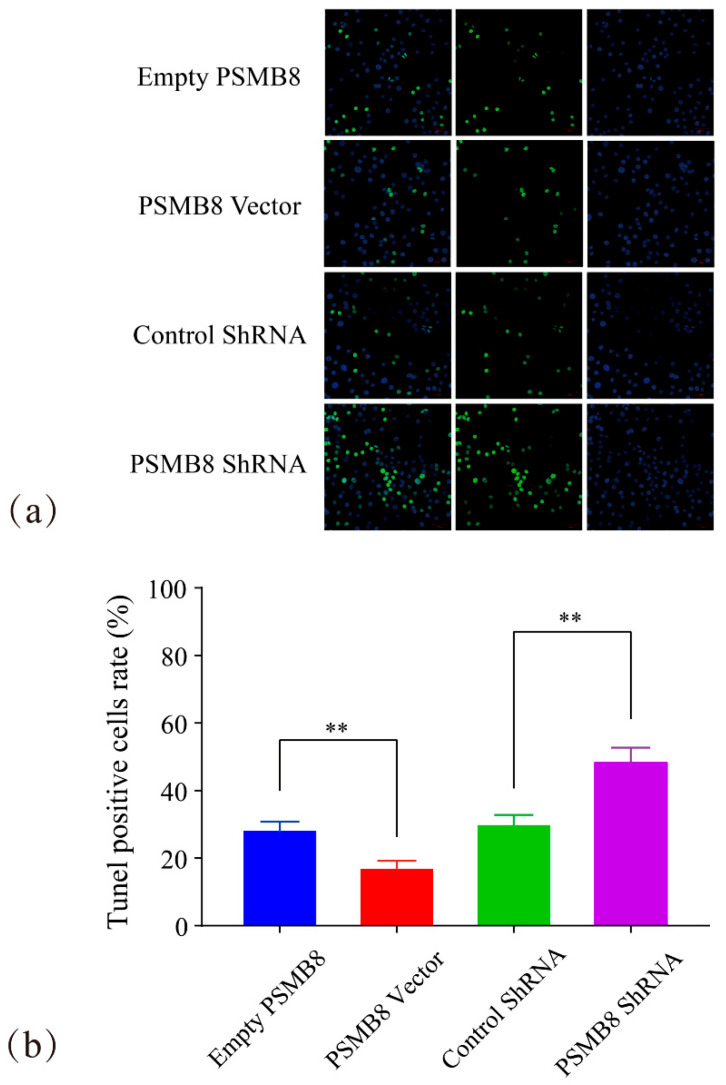
TUNEL assay for PSMB8-mediated HepG_2_ apoptosis. (**a**) TUNEL staining images of apoptotic cells in each PSMB8 group. In the TUNEL staining images, green fluorescent signals indicate TUNEL-positive apoptotic cells, and blue signals represent DAPI-stained cell nuclei. (**b**) Statistical analysis of TUNEL-positive cells. Data are presented as mean ± SD from three independent biological replicates (*n* = 3). Statistical analysis was performed using one-way ANOVA followed by LSD post-test. ** *p* ≤ 0.01.

Further verification was performed using the TUNEL assay (Figure 14). The TUNEL-positive cell ratio was 17.66% ± 1.53 in the PSMB8 Vector group. This value was highly significantly lower than that in the Empty PSMB8 group (28.96% ± 1.85, *p* ≤ 0.01). The TUNEL-positive cell ratio was 49.17% ± 3.51 in the PSMB8 shRNA group. This value was highly significantly higher than that in the Control shRNA group (30.59% ± 2.20, *p* ≤ 0.01).

These findings from the two assays were consistent. Inhibition of PSMB8 expression significantly enhanced apoptotic death in HepG_2_ cells.

### 3.6. Effects of Lingonberry Extract on HepG_2_ Cell Proliferation and Apoptosis

#### 3.6.1. Effect of Lingonberry Extract on PSMB8 Expression in HepG_2_ Cells

**Figure 15:**
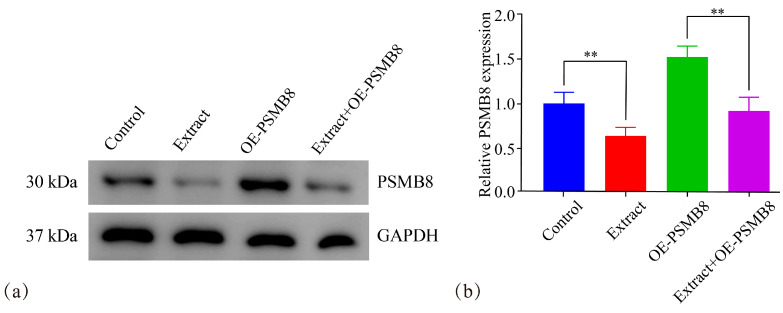
Western blotting analysis and densitometric quantification of PSMB8 protein expression in Lingonberry extract-treated cells. (**a**) Representative Western blotting bands of PSMB8 and GAPDH. (**b**) Densitometric quantification of PSMB8 protein levels normalized to GAPDH. Data are presented as mean ± SD from three independent biological replicates (*n* = 3). Statistical analysis was performed using one-way ANOVA followed by LSD post-test. ** *p* ≤ 0.01.

Western blotting analysis combined with densitometric quantification showed that PSMB8 protein expression was significantly lower in the lingonberry extract-treated group than in the corresponding untreated control group, regardless of PSMB8 overexpression (Figure 15).

**Figure 16:**
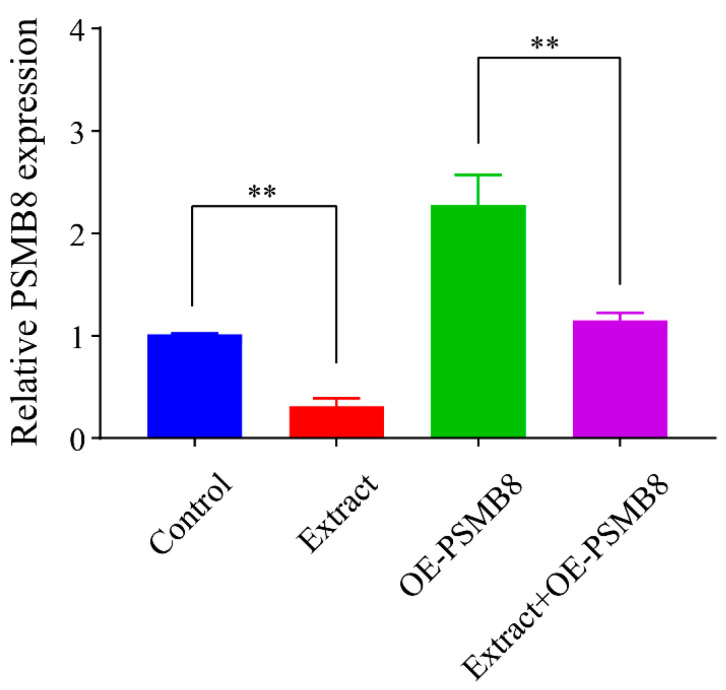
RT-PCR analysis of PSMB8 expression in Lingonberry extract-treated cells. Data are presented as mean ± SD from three independent biological replicates (*n* = 3). Statistical analysis was performed using one-way ANOVA followed by LSD post-test. ** *p* ≤ 0.01.

RT-PCR analysis further confirmed that lingonberry extract downregulated PSMB8 mRNA expression in both control and OE-PSMB8 cells (Figure 16).

These results suggest that lingonberry extract effectively inhibits PSMB8 expression in HepG_2_ cells.

#### 3.6.2. Effect of Lingonberry Extract on HepG_2_ Cell Proliferation via PSMB8 Regulation

**Figure 17:**
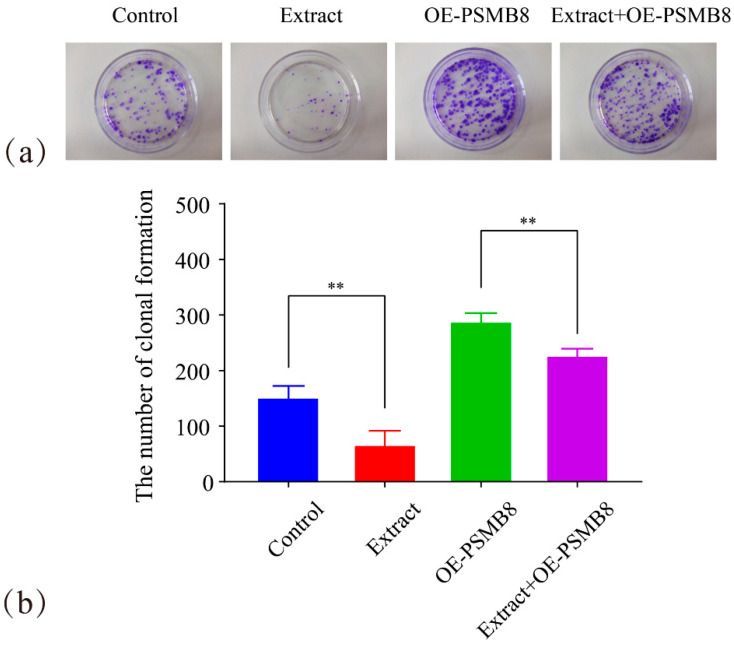
Effect of Lingonberry extract on HepG_2_ clonogenicity via PSMB8 regulation. (**a**) Representative images of cell clones in Lingonberry extract treatment groups. (**b**) Quantitative analysis of clonogenic significance. Data are presented as mean ± SD from three independent biological replicates (*n* = 3). Statistical analysis was performed using one-way ANOVA followed by LSD post-test. ** *p* ≤ 0.01.

A colony formation assay was used to investigate the regulatory role of Lingonberry extract in HepG_2_ cell proliferation. Its relationship with PSMB8 was also examined (Figure 17).

The Extract group formed 64 ± 27 colonies. This number was significantly fewer than that in the Control group (153 ± 19 colonies, *p* ≤ 0.01). The Extract+OE-PSMB8 group formed 225 ± 14 colonies. This number was significantly fewer than that in the OE-PSMB8 group (286 ± 17 colonies, *p* ≤ 0.01). These data indicated that Lingonberry extract significantly inhibited HepG_2_ cell proliferation. This inhibitory effect was mediated by targeting PSMB8.

HepG_2_ cells inherently show high PSMB8 expression. PSMB8 overexpression further enhanced cell proliferation. Lingonberry extract partially reversed this pro-proliferative effect. However, it could not fully restore colony numbers to the control level. Lingonberry extract still significantly suppressed HepG_2_ cell proliferation. These results confirmed that PSMB8 serves as a key target for Lingonberry extract in inhibiting HepG_2_ cell proliferation.

#### 3.6.3. Effect of Lingonberry Extract on HepG_2_ Cell Apoptosis via PSMB8 Regulation

**Figure 18:**
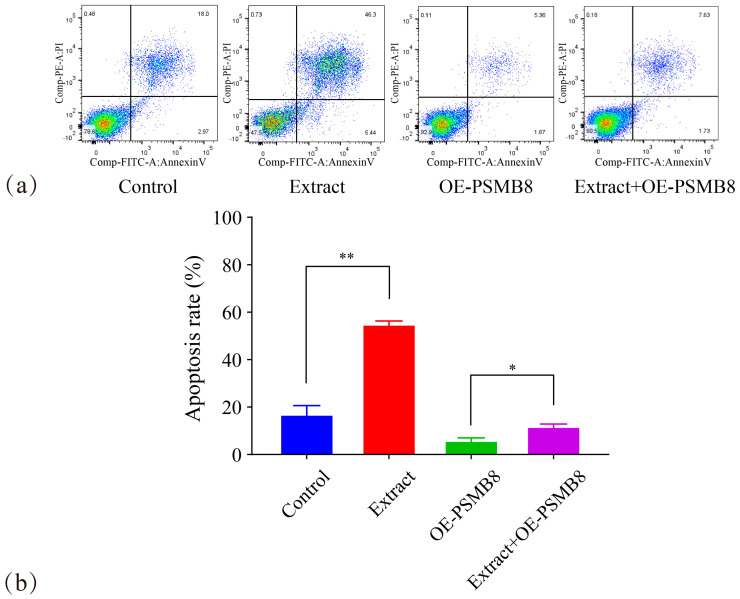
Effect of Lingonberry extract on HepG_2_ apoptosis via PSMB8 regulation. (**a**) Apoptosis assay results in Lingonberry extract treatment groups. In the flow cytometry dot plots, green fluorescent signals represent Annexin V-FITC staining and blue signals denote PI staining; color clusters distinguish cells at different apoptotic stages. (**b**) Statistical analysis of apoptotic cells. Data are presented as mean ± SD from three independent biological replicates (*n* = 3). Statistical analysis was performed using one-way ANOVA followed by LSD post-test. * *p* ≤ 0.05, ** *p* ≤ 0.01.

Flow cytometry was used to investigate two key aspects. It detected cell apoptosis levels and apoptotic stage distribution. It also explored the pro-apoptotic effect of Lingonberry extract on HepG_2_ cells and its association with PSMB8 (Figure 18).

The apoptotic rate of the Extract group was 54.03% ± 2.25. This value was highly significantly higher than that of the Control group (16.32% ± 4.27, *p* ≤ 0.01). The apoptotic rate of the Extract+OE-PSMB8 group was 11.16% ± 1.65. This value was significantly higher than that of the OE-PSMB8 group (5.23% ± 1.75, *p* ≤ 0.05). Notably, Lingonberry extract predominantly induced late apoptosis in HepG_2_ cells under both conditions.

HepG_2_ cells inherently exhibit high PSMB8 expression. PSMB8 overexpression further reduced the basal apoptotic rate. Lingonberry extract partially counteracted the effect of PSMB8 overexpression and restored apoptosis. However, it could not fully reach the apoptotic level of the Control group. Nevertheless, Lingonberry extract still significantly promoted HepG_2_ cell apoptosis. These results confirmed that PSMB8 is a key target through which Lingonberry extract induces late apoptosis in HepG_2_ cells.

## 4. Discussion

In this study, we employed a cross-species bioinformatics pipeline combined with in vitro experiments. We investigated the effects of Lingonberry extract on HepG_2_ cells and its association with PSMB8. Our findings identified PSMB8 as a pivotal target. We demonstrated that Lingonberry extract inhibits proliferation and induces late apoptosis in HepG_2_ cells. These effects are mediated by suppressing PSMB8 expression. These results suggest that PSMB8 mediates the pro-apoptotic effects of Lingonberry extract. This provides a mechanistic explanation for its anti-proliferative activity in HepG_2_ cells.

A major strength of this study is its stepwise bioinformatics screening strategy. Traditional HCC target screening is often hindered by host cell contamination in xenograft models. It is also limited by signal dilution due to tumour heterogeneity in clinical samples [28,29]. To address these challenges, we used cross-species transcriptome comparison and human-derived PPI network refinement. Two-dimensional GO-BP functional matching between human and mouse was also applied. By focusing on conserved immune-related biological processes, we improved the biological credibility of target selection. Furthermore, we integrated five distinct topological analysis approaches in Cytoscape. These approaches included MCODE, MNC, Betweenness, Bottleneck, and Clustering Coefficient. We identified core gene targets through intersection analysis of the resulting gene sets. Collectively, these analytical strategies effectively reduced interference from host–cell signals. They also improved the accuracy and reliability of target identification. Focusing on evolutionarily conserved functions enhances the translational potential of the identified targets. This systematic bioinformatics approach provides a reference for target identification in cross-species omics studies. It is also useful for screening in heterogeneous clinical samples. A potential limitation related to the bioinformatics pipeline should also be acknowledged. Xenograft RNA-seq reads were aligned to the mouse reference genome. Dual-genome alignment was not used, which may carry a risk of cross-species read misassignment. In addition, gene mapping based solely on sequence homology has limitations. It cannot fully ensure consistent functional conservation between human and mouse orthologs. However, our conserved-gene prioritization strategy was specifically designed. It did not aim to eliminate this misassignment risk. Instead, it focused on the functional conservation of genes across human and mouse species. This was applied even in the presence of mapping ambiguity. Potential uncertainties related to gene mapping and functional conservation were further mitigated. This was achieved through GO-BP functional matching and in vitro validation in human cell models. This approach allowed us to capture evolutionarily conserved targets. These targets would be inadvertently excluded by dual-genome alignment with strict species separation. Future studies may integrate dual-genome alignment as a complementary tool. This could further optimise mapping accuracy and analytical rigor.

In terms of translational potential, Lingonberry extract has multiple advantages. It is multi-component, multi-targeted, and exhibits low toxicity. Previous studies have shown that Lingonberry anthocyanins can alleviate liver fibrosis. They act by inhibiting hepatic stellate cell activation [30]. This suggests that Lingonberry extract may also improve the liver microenvironment. Our results supplement the anti-HCC mechanism of Lingonberry extract in HepG_2_ cells. They support its further development as a potential natural agent for HCC intervention. PSMB8 expression in HepG_2_ cells may serve as an indicator of sensitivity to Lingonberry extract. Notably, our results showed that Lingonberry extract only partially reversed the anti-apoptotic effect of PSMB8 overexpression. This implies that PSMB8 is a key but not the sole target mediating the anti-tumour effects of Lingonberry extract. This finding is consistent with the multi-component and multi-target nature of natural products. Further studies are needed to identify additional potential targets in this process.

The treatment of HCC still faces multiple clinical challenges [31,32]. The immunoproteasome subunit PSMB8 is highly expressed in HCC tissues. It is also closely associated with patient prognosis [33]. As a molecule involved in both immune regulation and apoptosis, PSMB8 represents a promising intervention target. Future studies are warranted to validate the regulatory effects of Lingonberry extract on PSMB8 in additional HCC cell models. This validation may help establish this natural product as a potential candidate or adjuvant agent for HCC intervention.

Nevertheless, several limitations of this study should be noted. First, the present study was performed only in HepG_2_ cells. The generalisability of our findings to other HCC cell lines remains to be confirmed. Second, in vivo experiments and clinical sample validation have not been performed. Third, the key active components in Lingonberry extract that contribute to PSMB8 regulation remain to be further identified and verified. Fourth, the detailed downstream signalling pathways of PSMB8 require further investigation. Future studies will focus on these aspects. This will help establish a more comprehensive regulatory network. It will also support the translational development of Lingonberry extract.

## 5. Conclusions

In conclusion, this study demonstrated that Lingonberry extract inhibits proliferation and induces apoptosis in HepG_2_ cells by downregulating PSMB8 expression. The cross-species bioinformatics strategy applied here improved the accuracy and reliability of target screening. These findings support the potential value of Lingonberry extract as a natural candidate for further study toward HCC intervention.

## Data Availability

The data presented in this study are openly available in NCBI Sequence Read Archive (SRA) at https://www.ncbi.nlm.nih.gov/sra/ accessed on 1 March 2026, reference number PRJNA1433823. The data will be publicly accessible upon publication of this manuscript.

## Supplementary Materials

The following supporting information can be downloaded at: https://www.mdpi.com/article/10.3390/cimb48030323/s1.
