# Supplementary material for: PSMB8 as a Core Target Mediating the Anti-Hepatocellular Carci-Noma Activity of Lingonberry (Vaccinium vitis-idaea L.) Extract in HepG2 Cells
Source: Curr Issues Mol Biol. 2026 Mar 18;48(3):323. doi: 10.3390/cimb48030323 (PMC13025438; doi:10.3390/cimb48030323)
Supplement: Supplementary file 1 [file cimb-48-00323-s001.zip › Supplementary Material S1.pdf]

Supplementary File S1

Characterization and optimal concentration of Lingonberry extract.

This supplementary material presents HPLC-based chemical characterization data of Lingonberry extract (from our previously published work, https://doi.org/10.1371/journal.pone.0270677), the optimal effective concentration of the extract in $HepG_2$ cells identified by CCK-8 assay, and batch-to-batch functional consistency verification of the extract via CCK-8 assay (conducted in this study) for experimental reliability.

1. Chemical characterization of Lingonberry extract

1.1. Identification Method of Lingonberry Extract

An Ultimate 3000 UPLC/HPLC system and Thermo Scientific Q Exactive were used for analysis of the composition of Lingonberry extract. Chromatography parameters were as follows: Hypersil Gold 100×2.1 mm, 3 μm column (Thermo Scientific, Germany) at 25 ℃; 0.1% acetic acid in acetonitrile (mobile phase A) and 0.1% acetic acid in water (mobile phase B) with gradient elution: A = 5–20–90–90–5–5% at 0–4–15–18–18.1–25 min; detection wavelength 280 nm; flow rate 0.25 mL/min. An m/z mass spectrometry scan was performed under electrospray ionization.

1.2. Lingonberry Extract Composition

These components were present in the following proportions（Tab.1 and Fig. 1）: 195.88 μg/mg cyanidin-3-O-glucoside chloride (37.58%), 57.13 μg/mg kaempferol 3-O-arabinoside (10.96%), 23.56 μg/mg epicatechin (4.52%), 22.67 μg/mg chlorogenic acid (4.35%), 19.96 μg/mg catechinic acid (3.83%), 8.03 μg/mg isoquercitrin (1.54%), 5.47 μg/mg 4-hydroxycinnamic acid (1.05%), 5.37 μg/mg cyanidin chloride (1.03%), 4.43 μg/mg 2,3-dihydroxybenzoic acid (0.85%), 2.87 μg/mg quercetin (0.55%), 1.88 μg/mg D-(-)-quinic acid (0.36%), 1.20 μg/mg caffeic acid (0.23%), 0.83 μg/mg ferulic acid (0.16%), 0.63 μg/mg oleanolic acid (0.12%), and 0.17 μg/mg ursolic acid (0.03%).

Table 1. Composition of Lingonberry Extract

number	Bioactive compound	Content (μg/mg)	Relative proportion (%)
1	Cyanidin-3-O-glucoside chloride	195.88	37.58
2	Kaempferol 3-O-arabinoside	57.13	10.96
3	Epicatechin	23.56	4.52
4	Chlorogenic acid	22.67	4.35
5	Catechinic acid	19.96	3.83
6	Isoquercitrin	8.03	1.54
7	4-Hydroxycinnamic acid	5.47	1.05
8	Cyanidin chloride	5.37	1.03
9	2,3-Dihydroxybenzoic acid	4.43	0.85
10	Quercetin	2.87	0.55
11	D-(-)-Quinic acid	1.88	0.36
12	Caffeic acid	1.2	0.23
13	Ferulic acid	0.83	0.16
14	Oleanolic acid	0.63	0.12
15	Ursolic acid	0.17	0.03

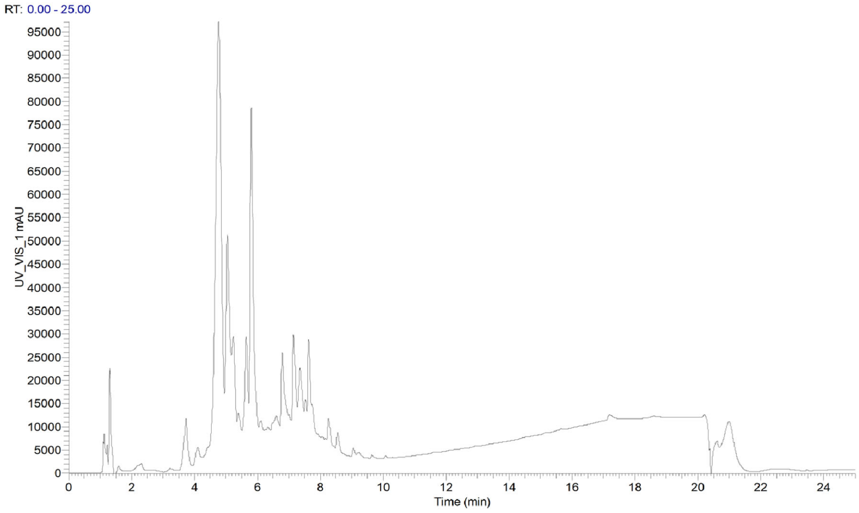

Figure 1. HPLC chromatogram of Lingonberry extract

Figure legend: HPLC chromatogram monitored at 280 nm, a characteristic wavelength for detecting the major phenolic compounds (anthocyanins and flavonoids) in Lingonberry extract.

2. Optimal concentration selection of Lingonberry extract

2.1. Cell Inhibition rate by CCK8 assay

Cells in logarithmic growth phase were inoculated in 96-well plates at a density of 1×10^4 cells/well and incubated overnight. After adherence, the old culture medium was replaced with varying concentrations of Lingonberry extract (10, 30, 50, 70, 90 μg/mL), while the control group received the same volume of culture medium. Following 24 h and 48 h of incubation, fresh culture medium and CCK8 assay solution were added and incubated for 2 h at 37°C to determine cell inhibition rates using enzyme markers. Absorbance values at 490 nm were measured using an enzyme counter after reintroducing fresh culture medium and CCK8 assay solution post-incubation.

2.2. Results

Inhibitory effect of Lingonberry extract on $HepG_2$ proliferation

Lingonberry extract was found to have an inhibitory effect on hepatocellular carcinoma $HepG_2$ cells after 24 h of treatment. The inhibitory effect on $HepG_2$ cells gradually increased with the concentration of Lingonberry extract ranging from 10 to 90 μg/mL (Fig. 2). At a concentration of 90 μg/mL, the inhibition rate of $HepG_2$ cells reached 52.32 ± 4.71%, with an IC_{50} value of 71.32 μg/mL.

After 48 h of treatment, the inhibitory effect of Lingonberry extract on $HepG_2$ cells continued to increase with concentration. The inhibition rates at 70 μg/mL and 90 μg/mL were 79.11 ± 2.88% and 81.21 ± 3.87%, respectively. Although the inhibition rate increased with concentration, there was no significant difference between the two concentrations. To conserve reagents, 70 μg/mL of Lingonberry extract was chosen as the appropriate concentration, with an IC_{50} value of 17.54 μg/mL.

The inhibition rate of Lingonberry extract on $HepG_2$ cells was observed to be less than 60% within the concentration range of 10 to 90 μg/mL after 24 h of exposure, with no acute toxicity detected. After 48 h, the inhibition rate increased with concentration, significantly reducing cell proliferation in the Lingonberry extract group compared to the control group. This indicates that Lingonberry extract

effectively inhibits $HepG_2$ cell growth.

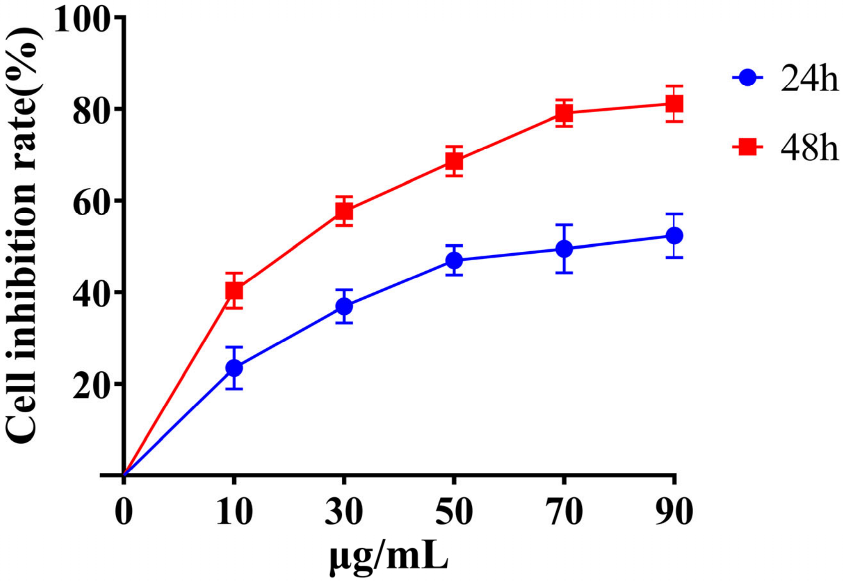

Figure 2. Inhibitory effect of Lingonberry extract on $HepG_2$ cell proliferation
